# Photocatalytic Perfomance of ZnO-Graphene Oxide Composites towards the Degradation of Vanillic Acid under Solar Radiation and Visible-LED

**DOI:** 10.3390/nano11061576

**Published:** 2021-06-15

**Authors:** Neda Mirikaram, Álvaro Pérez-Molina, Sergio Morales-Torres, Amir Salemi, Francisco J. Maldonado-Hódar, Luisa M. Pastrana-Martínez

**Affiliations:** 1 Department of Inorganic Chemistry, Faculty of Sciences, University of Granada, Avda. Fuente Nueva s/n, ES-18071 Granada, Spain; mirikaram@gmail.com (N.M.); alpemo@ugr.es (Á.P.-M.); semoto@ugr.es (S.M.-T.); fjmaldon@ugr.es (F.J.M.-H.); 2 Environmental Sciences Research Institute, Shahid Beheshti University, Tehran 19839-63113, Iran; a_salemi@sbu.ac.ir

**Keywords:** graphene oxide, ZnO, vanillic acid, water treatment, scavengers

## Abstract

Graphene oxide (GO) is used to enhance the photocatalytic activity of ZnO nanoparticles for the degradation of vanillic acid (VA) under simulated solar light and visible-LED (*λ* > 430 nm). ZnO-GO composites are prepared by a mixing and sonication process with different GO loadings (i.e., from 1.8 to 6.5 wt.%). The materials are extensively characterized by thermogravimetric analysis (TGA), physisorption of N_2_, X-ray diffraction (XRD), infrared spectroscopy (FTIR), scanning electron microscopy (SEM), point of zero charge (pH_PZC_), and UV-Vis diffuse reflectance spectroscopy (DRUV). The presence of GO increases the photocatalytic activity of all the prepared composites in comparison with the pristine ZnO. The highest photocatalytic activity is found for the composite containing 5.5 wt.% of GO (i.e., ZnO-GO5.5), reaching a VA degradation of 99% and 35% under solar light and visible-LED, respectively. Higher TOC removal/VA degradation ratios are obtained from the experiments carried out under visible-LED, indicating a more effective process for the mineralization of VA than those observed under simulated solar light. The influence of hole, radical, and non-radical scavengers is studied in order to assess the occurrence of the reactive oxygen species (ROS) involved in the photocatalytic mechanism. The study of the photo-stability during three reuse experiments indicates that the presence of GO in the composites reduces the photocorrosion in comparison with pristine ZnO.

## 1. Introduction

Over the past 40 years, advanced oxidation processes (AOPs) have been successfully applied to manage the problematic issues associated to water, air, and soil pollution [1]. These processes are mainly based on the generation of hydroxyl radicals (HO^•^) which can generate a sequence of reactions capable of degrading contaminants into CO_2_ and H_2_O, or converting them into less toxic organic compounds. These processes include heterogeneous photocatalysis, which is based on the effective utilization of ultraviolet (UV) or solar irradiation as a technology for environmental applications [2]. On the other hand, many structural materials including polymers, metals, ceramics, glasses, and composite materials have been synthetized for different industrial applications [3,4,5,6,7,8].

Normally, metal oxides such as titanium dioxide (TiO_2_), zinc oxide (ZnO), zirconia (ZrO_2_), tungsten trioxide (WO_3_), and vanadium oxide (V_2_O_5_), have been used as semiconductor photocatalysts for water and air decontamination, as well as for energy applications [9,10,11,12]. ZnO is an n-type semiconductor with a band gap ca. 3.22 eV and a large excitation binding energy of ca. 60 meV. It can be considered as a suitable successor of the benchmark TiO_2_ semiconductor due to its similar properties such as strong oxidation ability, good photocatalytic properties, chemical stability, biocompatibility, non-toxicity, high photosensitivity, and electronic and piezoelectric properties, among others [13,14,15]. This semiconductor usually exists in one-dimensional (1D), two-dimensional (2D) and three-dimensional (3D) associations [16], and has been used in a wide range of applications, such as sensors [17], photocatalysis [14,18,19,20,21], transistors, solar cells [22,23], etc. It is well known that the use of ZnO in photocatalysis displays some drawbacks such as: (i) the limitation of its use in the visible range due to its wide band gap [24]; (ii) particle aggregation during photocatalytic reactions which significantly restrict the photocatalytic activity of ZnO at a large scale [25]; and (iii) the rapid recombination of the photogenerated electron-hole pairs [26,27].

Many strategies have been carried out to improve the photocatalytic performance of ZnO, e.g., the design of suitable sizes and morphologies [28,29], noble metal loading [14,30,31], heteroatom doping [32,33,34], or by forming semiconductor composites [35], among others. In particular, the design and development of hybrids based on the coupling of carbon materials and ZnO is an effective option for enhancing its photocatalytic response under UV/Vis irradiation [18,36,37,38], by inhibiting the electron-hole recombination as well as extending the light absorption into visible range [39].

Amongst the vast number of carbon nanomaterials, graphene and its derivatives have been shown to significantly enlarge the photocatalytic degradation activity of ZnO in the field of photocatalytic wastewater treatment [37,40,41,42,43]. Of particular interest is the use of one of the most well-known derivatives of graphene, i.e., graphene oxide (GO), for the synthesis of ZnO-GO hybrid materials, as the presence of oxygen-containing surface groups in GO offers an excellent dispersibility in polar solvents for the synthesis of the composites. Furthermore, GO can improve the photocatalytic efficiency in the composites by: (i) acting as an electron-acceptor, hindering the electron-hole recombination; (ii) increasing the adsorption of organic pollutants through π-π interactions between the *sp^2^* region of graphene and the aromatic pollutant; and (iii) creating oxygen vacancies in the lattice of ZnO, extending its response under the visible light region [35,37,42,44,45]. Various methods have been reported for the synthesis of ZnO-GO composites, such as hydrothermal process [46,47], chemical vapor deposition (CVD) [35,48,49], physical vapor deposition (PVD) [50,51], pulsed laser deposition (PLD) [52], spray pyrolysis [53,54], solvothermal [19], and microwave synthesis [55], among others.

In this paper, we report a low-cost and scaling up method for the synthesis of ZnO-GO composites. Nanostructured ZnO particles were formed using zinc acetate as precursor, then the composites were synthetized by a simple mixing and sonication method. The photocatalytic performance of the prepared materials was tested in the degradation of a phenolic compound, namely vanillic acid (VA), under both simulated solar irradiation and visible-LED (*λ* > 430 nm). VA is a model compound typically present in the phenolic fractions of olive mill wastewater (OMW). The presence of this phenolic compound constitutes an important environmental problem, especially in Mediterranean countries and, in particular, areas of southern Spain, where a great number of plants are involved in the production and refining of olive oil [56].

## 2. Materials and Methods

### 2.1. Synthesis of GO, ZnO and ZnO-GO Composites

Graphite oxide was prepared using a modified Hummers’ method [57,58]. Specifically, 5 g of graphite (powder < 20 μm, Sigma–Aldrich, St. Louis, MO, USA) and 5 g of sodium nitrate, NaNO_3_ (99.0%, Acros Organics, Geel, Belgium) were put into 240 mL of concentrated sulfuric acid, H_2_SO_4_ (96–99%, supplied by PanReac AppliChem, Darmstadt, Germany). The mixture was kept stirring for 30 min in an ice bath to prevent the temperature exceeding 10 °C. Thereafter, 30 g of potassium permanganate, KMnO_4_ (99.0%, PanReac AppliChem, Darmstadt, Germany) was added gradually with stirring (30 min). The ice bath was then removed, and the mixture was warmed at 35 °C and stirred for 24 h. The reaction was terminated by slowly adding 800 mL of distilled water and 30 mL of 30% hydrogen peroxide, H_2_O_2_ (30% *w*/*w*, PanReac AppliChem, Darmstadt, Germany) solution. Finally, the resulting dispersion was filtered and washed repeatedly with distilled water until its pH became neutral. The acquired solid (i.e., graphite oxide) was dried in an oven at 60 °C. Graphene oxide (GO) was prepared by sonication in an aqueous solution using an ultrasound bath (ultrasonic processor UP400S, 24 kHz, Hielscher, Germany). The undissolved solid was removed by centrifugation for 30 min at 3000 rpm to obtain the GO suspension (1 g L^−1^).

ZnO nanoparticles were prepared by calcination of zinc acetate dihydrate, Zn(Ac)_2_ 2H_2_O (≥99.0%, Alfa Aesar, Haverhill, MA, USA) in a furnace with air flow at 5 °C min^−1^ until 600 °C, with a soak time of 3 h [59]. ZnO-GO composites were synthesized by a sonication method [38,60]. Briefly, the required amount of ZnO was added to different amounts of an aqueous GO dispersion (1 g L^−1^) and ethanol (96% *v*/*v*) mixture in a proportion of 1:2, respectively. The resulting dispersion was kept under vigorous agitation for 30 min, then sonicated for 2 h in an ultrasound bath (ultrasonic processor UP400S, 24 kHz, Hielscher, Germany). The obtained composites were filtered, washed with ultrapure water, and dried at 120 °C in a vacuum oven for 8 h. The GO loading was fitted at ca. 2, 3, 5, and 7 wt.%.

### 2.2. Characterization Techniques

Infrared spectra (ATR-IR) were recorded in a NICOLET 510P spectrometer (Thermo Fisher Scientific, Waltham, MA, USA) with an attenuated total reflection accessory and a ZeSn as ATR crystal. Thermogravimetric (TG) analysis of the composites was obtained using a SHIMADZU TGA–50H thermobalance (Shimadzu Corporation, Japan) by heating the sample in air flow up to 950 °C with a heating rate of 20 °C min^−^^1^. The N_2_ adsorption–desorption isotherms at −196 °C were obtained using a Quadrasorb SI equipment (Quantachrome, Boston Beach, FL, USA). The samples were outgassed overnight at 110 °C under high vacuum (10^−^^6^ mbar). The Brunauer–Emmett–Teller (BET) equation was applied to calculate the apparent surface area (S_BET_) [61,62], while the mesopore volume (V_meso_) and total pore volume (V_total_) were determined by applying the Barrett, Joyner, and Halenda (BJH) method [63,64] to the desorption branch of the N_2_ isotherms. The point of zero charge (pH_PZC_) of the materials was determined following the method described elsewhere [65,66]. The surface morphology of the synthesized photocatalysts was investigated by scanning electron microscopy (SEM) using a LEO (Carl Zeiss) GEMINI–1430–VP microscope (Oberkochen, Germany). Transmission electron microscopy (HRTEM) images were taken using a FEI Titan G2 60–300 microscope (FEI, Hillsboro, OR, USA) with a high brightness electron gun (X-FEG) operated at 300 kV and equipped with a Cs image corrector (CEOS). The X-ray diffraction (XRD) patterns were obtained using a Philips PW 1710 diffractometer (Bruker, Rivas-Vacia, Madrid, Spain) provided with a CuKα radiation and a nickel filter that removes κ*β* radiation. The average crystal size (D) of the samples was calculated by the Scherrer formula [67]:

(1) $$D=\frac{0.94\lambda }{\beta \cos\theta }$$

where *λ* is the wavelength of the X-ray, *β* is the FWHM (full-width at half-maximum) and *θ* is the diffraction angle. X-ray photoelectron spectroscopy (XPS) measurements were carried out using a Physical Electronics VersaProbe II apparatus (PHI, Chanhassen, MN, USA) equipped with a MgK_α_ X-ray source (*hν* = 1486.6 eV) operating at 1.3 V and 20 mA, and a hemispherical electron analyzer. Survey and multi-region spectra were recorded at the O1s and Zn2p photoelectron peaks. The optical properties of the photocatalysts were characterized by a UV–Vis spectrophotometer CARY 5E (VARIAN, Palo Alto, California, USA) equipped with a diffuse reflectance accessory (DRA. The band gap of the materials was calculated from the corresponding Tauc plots using (Abs·hν)^1/2^ units as a function of energy (eV).

### 2.3. Photocatalytic Tests

The photocatalytic performance of the prepared catalysts was evaluated for the degradation of vanillic acid, VA (C_8_H_8_O_4_, 97%, Sigma-Aldrich, St. Louis, MO, USA) in aqueous solutions under both simulated solar irradiation and visible-LED at room temperature (average 25 °C). Solar irradiation was carried out using a SOLAR BOX 1500e (CO.FE.MEGRA, Milano, Italy) with a 1500 W Xenon lamp (500 W m^−2^ of irradiance power). Irradiation with visible light was performed with an LED lamp from Oriel, model LSH-7320 LED Solar Simulator (Metrohm, Herisau, Switzerland), with a total power output of 110 mW cm^−2^ and a wavelength range from 410 to 1100 nm.

The photocatalytic experiments were performed in a glass reactor loaded with 50 mL of solution containing the model pollutant VA (20 mg L^−1^). The composite concentration was fixed as 1 g L^−1^ to avoid the effect of light scattering. The suspension was magnetically stirred and continuously purged with an oxygen flow. A dark period (30 min) was maintained before switching on the lamp in order to achieve the adsorption–desorption equilibrium conditions.

A syringe polyethersulfone (PES) filter of 0.45 µm (Agilent Technologies, CA, USA) was used to separate the photocatalyst from the solution. The concentration of VA was determined by Ultra High-Performance Liquid Chromatography (UHPLC), using a Shimadzu Corporation apparatus (model Nexera, Tokyo, Japan) equipped with a Pump LC-30AD, an Autosampler SIL-30AC, an Oven CTO-20AC, a Degasser DGU-20A5r, a System Controller CBM-20 A Lite, and a Diode Array Detector (SPD-M20A). Chromatographic separation was optimized using a Shim-pack GISS-HP C18 3 µm column (100 × 3.0 mm I.D.) supplied by Shimadzu Corporation (Tokyo, Japan). The temperature of the column oven and autosampler were set at 40 °C and 15 °C, respectively, while the injection volume was 20 µL. The mobile phase consisted of a mixture of acetonitrile, water, and acetic acid (29:70:1), respectively, at isocratic conditions and with a flow rate of 1 mL min^−^^1^.

The total organic carbon (TOC) content of initial and final samples was determined at the end of the experiments (i.e., 60 and 180 min for simulated solar light and visible-LED, respectively) using a TOC–5000A apparatus (Shimadzu, Kyoto, Japan).

The photocatalytic degradation was calculated using the following equation:

(2) $$\left[VA\right]={\left[VA\right]}_{0\;}\times e^{-k_{ap}\times t}$$

where *k_ap_* is the pseudo–first order kinetic constant, *t* is the reaction time, and [*VA*]_0_ and [*VA*] denote the pollutant concentration at *t* = 0 and *t* = t, respectively. The values of *k_ap_* were obtained by non–linear regression.

The photocatalytic degradation pathway of VA was studied using ethylenediaminetetraacetic acid (EDTA, 1.0 mM), furfuryl alcohol (FFA, 1.0 mM), and methanol (MeOH, 1.0 mM) and as hole, singlet oxygen (^1^O_2_), and radical scavengers, respectively [58].

## 3. Results and Discussion

### 3.1. Materials Characterization

**Figure 1:**
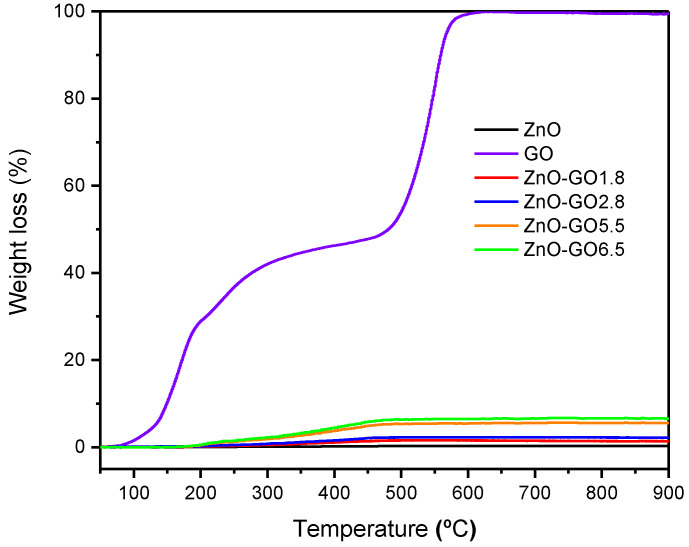
Thermogravimetric analysis in air of GO, pristine ZnO, ZnO-GO1.8, ZnO-GO2.8, ZnO-GO5.5, and ZnO-GO6.5 composites.

The GO content in the composites was verified by thermogravimetric analysis (TG). Figure 1 shows the thermogravimetric analysis (TG) under air flow for GO and ZnO, as well as for the composites. The GO content of the composites was determined by burning in TG experiments, which directly analyze the weight loss along the combustion of GO in the composite (Figure 1). The results indicate a weight loss of 1.8, 2.8, 5.5, and 6.5 wt.% which is in agreement with the nominal GO content of the composites (i.e., 2.0, 3.0, 5.0, and 7.0 wt.%., respectively). Taking into account the obtained weight loss, the composites were labelled as ZnO-GO1.8, ZnO-GO2.8, ZnO-GO5.5, and ZnO-GO6.5 for GO loading of 1.8, 2.8, 5.5 and, 6.5 wt.%, respectively. For GO, the weight loss observed up to ca. 500 °C should be attributed to the removal of oxygenated surface groups of GO, followed by the carbon combustion, as previously reported in the literature. [58,68]. Regarding the TG of composites, the carbon combustion occurs at lower temperatures (i.e., <500 °C). These results suggest that the presence of ZnO in the composites could have a catalytic effect on the combustion of the GO.

**Figure 2:**
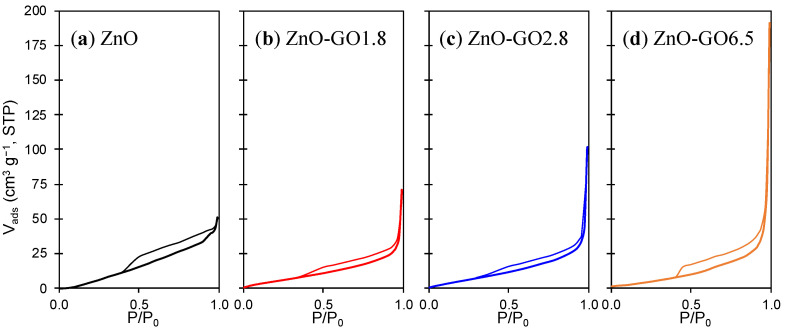
N_2_ adsorption–desorption isotherms of (**a**) pristine ZnO, (**b**) ZnO-GO1.8, (**c**) ZnO-GO2.8 and (**d**) ZnO-GO6.5.

The textural characterization of pristine ZnO and the corresponding composites was studied by physisorption of N_2_ at −196 °C. Figure 2 shows the N_2_ adsorption-desorption isotherms of the prepared materials. In general, the isotherms can be classified as type-II, in accordance with IUPAC classification. These results indicate the samples consist of macroporous materials, or materials with a low porosity [60]. In fact, the N_2_ volume is negligible as a consequence of the absence of micropores at a very low relative pressure. However, a large amount of N_2_ is adsorbed at high relative pressures due to the presence of large mesopores. In addition, all isotherms presented a small hysteresis loop of type H3, typical of agglomerates formed by platelets or adsorbents with slit-shaped pores, which could correspond to GO layers coated by ZnO particles.

Apparent surface areas (*S*_BET_) of all prepared materials were between 10 and 19 m^2^ g^−1^, with ZnO-GO composites usually presenting higher *S*_BET_ than pristine ZnO (e.g., 12 and 18 m^2^ g^−^^1^ for pristine ZnO and ZnO-GO5.5, respectively, Table 1). In general, the addition of GO improved the porosity of the composites, namely the mesoporore volume (*V*_meso_) and the total pore volume (*V*_total_), these parameters generally increasing as the GO loading increased (e.g., *V*_total_ = 0.10 and 0.29 cm^3^ g^−1^ for ZnO-GO1.8 and ZnO-GO6.5, respectively). This improvement in composites’ porosity should be attributed to the intercalation of GO layers with ZnO particles generating new interstitial spaces in the mesopore range.

The pH_PCZ_ for ZnO, GO and the composites are listed in Table 1. The pH_PZC_ value of GO was 2.9, indicating the strong acidic character of the GO surface due to the presence of a large amount of oxygenated groups (mainly epoxy and hydroxyl groups) [69]. pH_PZC_ could change depending on the synthesis method and structure of ZnO [70]. In this study, the pH_PZC_ for ZnO was calculated to be approximately 7.6, indicating the neutral/slightly basic character of the semiconductor. The results point out that the pH_PZC_ values for ZnO-GO composites decrease as the GO content increases, resulting in materials with a slightly lower basic character than pristine ZnO [69].

**Figure 3:**
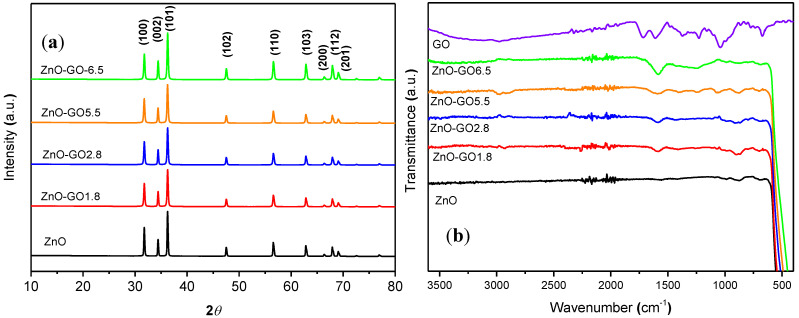
(**a**) XRD patterns of ZnO and ZnO-GO composites, (**b**) FTIR spectra of GO, ZnO and ZnO-GO composites.

Figure 3a shows the XRD patterns of ZnO nanoparticles and the ZnO-GO composites. The similar patterns obtained denote that the crystalline structure of bare ZnO is maintained in the composites, as reported elsewhere [37,42]. Peaks observed at 31.9°, 34.5°, 36.3°, 47.6°, 56.6°, 62.9°, 66.5°, 68.0°, and 69.3° correspond to the (100), (002), (101), (102), (110), (103), (200), (112), and (201) planes of the hexagonal ZnO wurtzite structure (JCPDS No. 36-1451), respectively [35,37]. Moreover, the characteristic peak of GO at approximately at 12°, and associated to the reflection for the (001) plane of GO, is not observed in the XRD patterns, due to the low content of GO present in the composites, as well as to the strong diffractions of ZnO that could mask the peak associated to GO [42,44]. The particle size of the ZnO nanoparticles and the ZnO composites were calculated using the Scherrer’s equation and the results are included in Table 1. Particles sizes of 41, 36, 34, 35, and 35 nm were calculated for ZnO, ZnO-GO1.8, ZnO-GO2.8, ZnO-GO5.5, and ZnO-GO6.5 photocatalysts, respectively. In general, all the materials showed similar particles size values.

ATR-IR spectra of pristine ZnO, GO and ZnO-GO composites are depicted in Figure 3b. GO spectrum shows the main characteristic bands associated to the presence of oxygen functionalities at around 1050, 1350, and 1720, and a broad band ca. at 3000–3400 cm^−^^1^ attributed to C–O, C–OH (stretching), C=O, and C–OH (vibration) groups, respectively [64]. The spectra of ZnO and the composites show a common high intensity band at around 450–500 cm^−^^1^, corresponding to the stretching vibration of Zn-O [71]. In general, the IR-spectra of the ZnO-GO composites also show two weak bands at around 1600 cm^−^^1^ and 850 cm^−^^1^, attributed to the bending vibration of water and to the Zn–OH group, respectively [71,72,73]. It is interesting to note that the intensity of the peaks associated to carbonyl groups (C=O) and epoxy groups (C–O) at around 1700 and 1100 cm^−^^1^, respectively, decreased significantly for the ZnO-GO composites. These results indicate that the anchoring of ZnO to GO could be preferentially through these groups.

**Figure 4:**
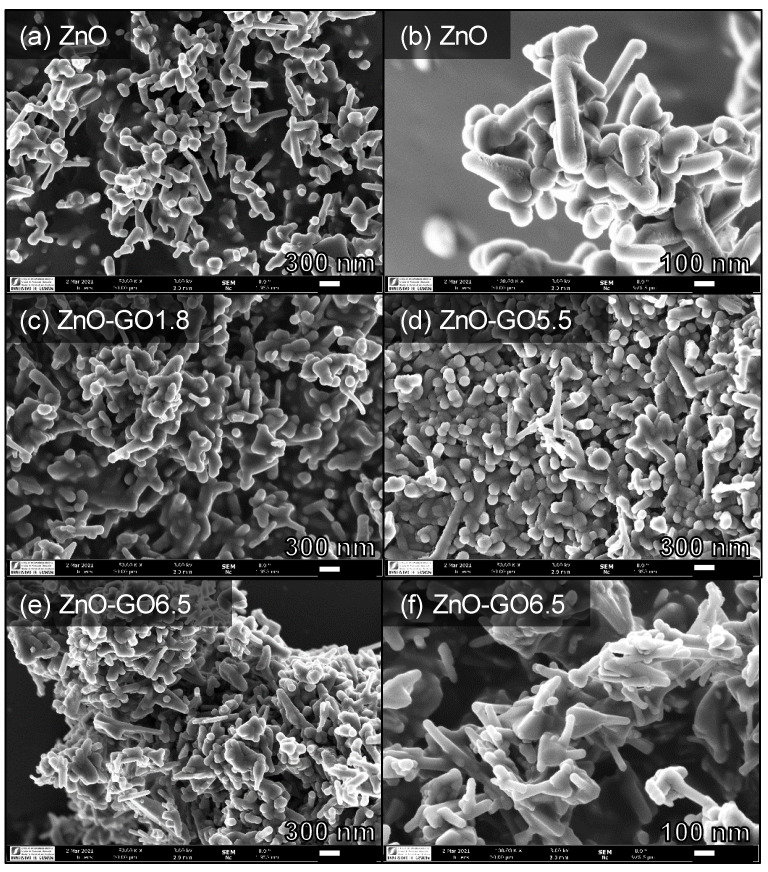
SEM micrographs of (**a**,**b**) ZnO, (**c**) ZnO-GO1.8, (**d**) ZnO-GO5.5, and (**e**,**f**) ZnO-GO6.5.

The SEM images of ZnO and ZnO-GO1.8, ZnO-GO5.5, and ZnO-GO6.5 are depicted in Figure 4a–f (ZnO and ZnO-GO6.5 are shown for two different magnifications). The ZnO structure consists of rod-like particles with size of ~100 nm and spherical-like particles with ca. 50 nm of diameter (Figure 4a,b). In general, ZnO-GO composites show more aggregated structures in comparison with pristine ZnO, originating larger particle clusters and progressively favouring the formation of flat structures. The formation of ZnO nanoparticles from acetate/nitrate, forming from nanorods to nanoflakes, or grouped forming flower-like structures, depend on the experimental conditions, as previously described [72]. The composite micrographs (Figure 4c–f) do not show GO sheets uncoated with ZnO, indicating a good assembly between ZnO and GO phases for all the composites prepared.

**Figure 5:**
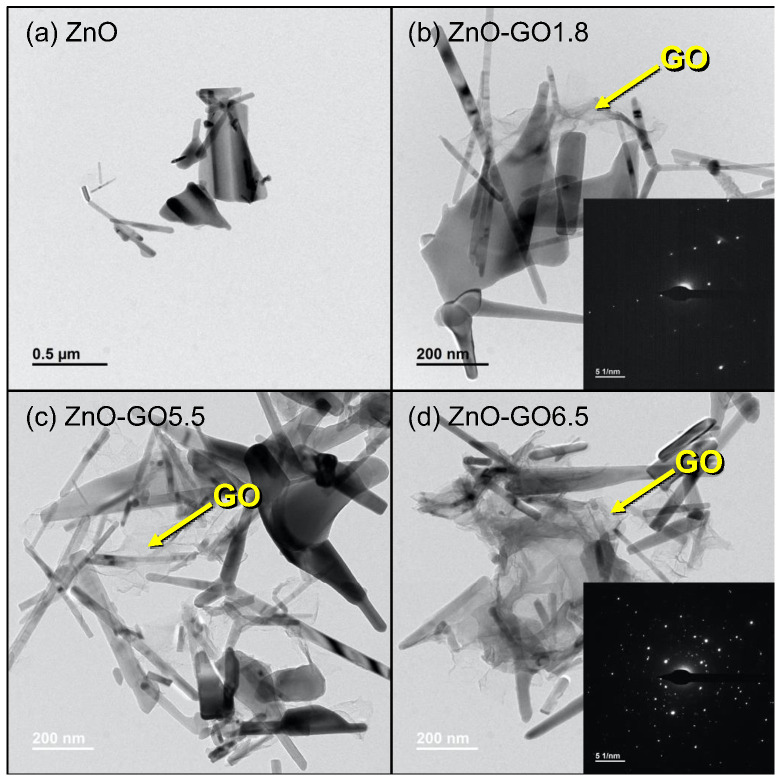
HRTEM micrographs of (**a**) ZnO, (**b**) ZnO-GO1.8, (**c**) ZnO-GO5.5, and (**d**) ZnO-GO6.5. Selected areas electron diffraction (SAED) images are included as insets.

Figure 5 shows HRTEM images of pristine ZnO and several ZnO-GO composites. In general, ZnO consists of rod-like nanoparticles with a uniform size distribution and clear crystalline structure. Regarding the ZnO-GO composites, micrographs reveal the presence of GO in the composites in the form of aggregated sheet-like structures. The HRTEM images (Figure 5b–d) also show a uniform distribution of the GO between the ZnO structures, inducing a good contact between the two phases. SAED images for selected samples allowed us to corroborate the polycrystalline character of ZnO particles with some of them highly ordered (inset—Figure 5b), and others with several overlapping planes (hkl) corresponding to different nanocrystals (inset—Figure 5d).

**Figure 6:**
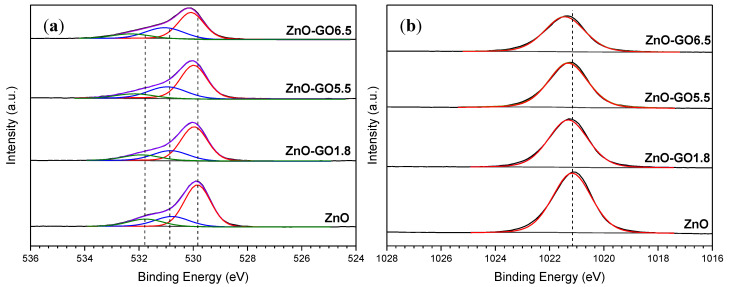
XPS spectra and deconvolution of (**a**) O1s and (**b**) Zn2p regions of ZnO and selected ZnO-GO composites. (The Zn2p_3/2_ region was only represented for clarification).

The chemical composition of ZnO and the ZnO-GO composites was studied by XPS, the results corresponding to the analysis for the O1s and Zn2p regions shown in Figure 6a,b, respectively. The O1s spectra of the materials were deconvoluted into three components, the first peak placed at ~529.8 eV is assigned to O_2_^−^ ions from Zn–O bonds belonging to the ZnO wurtzite structure, while the second one at ~530.8 eV corresponds to OH groups absorbed onto the ZnO surface [74], and double-bonded oxygen (C=O) of oxygen-containing groups anchored in the GO structure [75]. The last peak of the O1s region located ~531.8 eV can be ascribed to single-bonded oxygen (C–O) from the oxygen functionalities of GO, in particular alcohol, ether, and epoxy groups [75]. The detected carbon for the ZnO sample could be related to the carbon adsorbed on its surface during the exposure of the sample to the ambient atmosphere [74].

Regarding the Zn2p region, two peaks were located at ~1021.2 and ~1044.2 eV, attributed to Zn2p_3/2_ and Zn2p_1/2_, respectively, and a binding energy (B.E.) difference of 23.0 eV [74]. The XPS spectra of Zn2p region for the different materials only showed a clear peak at ~1021.2 eV, which denotes a Zn^2+^ oxidation state. For ZnO-GO composites with larger GO contents, a shifted Zn2p_3/2_ peak towards a higher B.E was observed, due to the chemical environment interaction between ZnO particles and GO functionalities.

**Figure 7:**
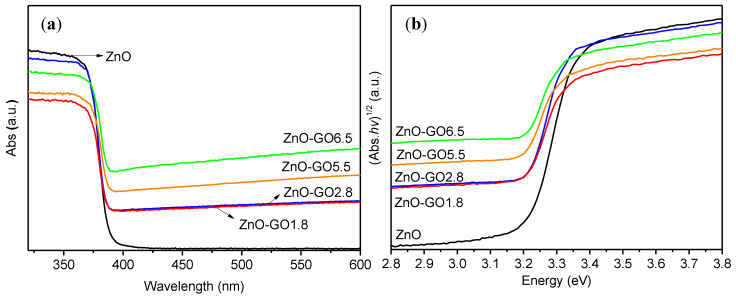
(**a**) UV–vis spectra and (**b**) Tauc’s plots versus the energy in eV of ZnO and ZnO-GO composites.

UV–Vis diffuse reflectance spectra were carried out in order to determine the electronic properties of pristine ZnO and their composites. For all the photocatalysts, a strong intense absorption band in the UV range with onset at <400 nm was observed (Figure 7a) This band is associated to the intrinsic band-gap absorption of pristine ZnO [35]. It can be seen that the absorption intensity of the composites is significantly improved in the visible region due to the presence of GO [35,37,44]. In general, this effect is proportional with the different GO loading on the composites, obtaining a stronger absorption capacity in the visible region for the composites with a higher amount of GO (i.e., ZnO-GO5.5 and ZnO-GO6.5). This effect can be attributed to the capacity of carbon materials to absorb light as well as to the creation of electronic interactions between carbon and ZnO as reported in the literature for the case of other carbon and metal oxide phases [38,60,76]. Figure 7b shows the Tauc’s plots versus the energy (eV). The calculated E_g_ of ZnO, ZnO-GO1.8, ZnO-GO2.8, ZnO-GO5.5, and ZnO-GO6.5 were 3.12, 3.05, 3.05, 2.98, and 2.95 eV, respectively (Table 1), being the obtained band gap for the composites lower than the value obtained for pristine ZnO.

### 3.2. Photocatalytic Activity of the ZnO-GO Composites

**Figure 8:**
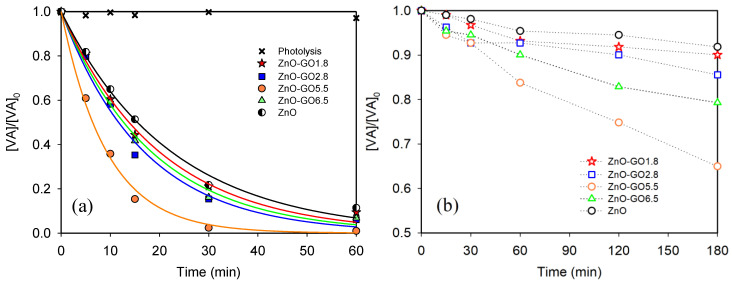
Photocatalytic degradation of VA as a function of time for ZnO and the ZnO-GO composites under (**a**) simulated solar light and (**b**) visible-LED.

The photocatalytic efficiency of ZnO and ZnO-GO composites (with different GO loading) for VA degradation under simulated solar light and visible-LED are shown in Figure 8a,b, respectively. The kinetic rate constant for solar light (*k_ap_*), the VA conversion (*X_VA_* (%)), and TOC removal (*X_TOC_* (%)) for both solar light and visible-LED are gathered in Table 2. The experiment in the absence of a photocatalyst (i.e., photolysis) shows a null degradation of the contaminant under both solar light (Figure 8a) and visible-LED (not shown). On the other hand, the adsorption equilibrium in dark conditions was established after 60 min for ZnO and ZnO-GO composites, with obtained values of approximately 3–8% of the initial VA concentration. For all the photocatalysts tested, 60 min was proven to be enough to reach the adsorption equilibrium.

Figure 8a shows a significant photocatalytic performance using all the prepared materials for the degradation of VA under solar light. The results indicate that the presence of GO in the composites enhances the degradation efficiency of pristine ZnO under solar light (*k_ap_ =* 100.8 × 10^−^^3^ min^−^^1^, 59.4 × 10^−^^3^ min^−^^1^, 54.6 × 10^−^^3^ min^−^^1^, 50.2 × 10^−^^3^ min^−^^1^, and 44.7 × 10^−^^3^ min^−^^1^ for ZnO-GO5.5, ZnO-GO2.8, ZnO-GO6.5, ZnO-GO1.8, and ZnO, respectively). Regarding the results obtained under visible-LED (Figure 8b), a similar trend was observed for VA degradation, i.e., ZnO-GO5.5 (35%) > ZnO-GO6.5 (20.7%) > ZnO-GO2.8 (14.4%) > ZnO-GO1.8 (9.9%) > ZnO (8.1%), where the values in brackets are the VA conversion (Table 2). As expected, the VA degradation under visible-LED was lower than that obtained under simulated solar irradiation as there is no UV irradiation, and only photons with *λ* > 430 nm can reach the samples. For all the photocatalysts tested, the results indicate that the presence of GO enhances the photocatalytic activity under both solar irradiation and visible-LED as previously reported [42,44,77,78]. The possible reasons for higher photocatalytic activity of the ZnO-GO composites compared to pristine ZnO could mainly be ascribed to the increased adsorption capacity, as well as the extended light absorption in the visible range (Figure 7a) [77,78]. Moreover, considering the conduction band of pristine ZnO (c.a. −4.05 eV) [79] and work function of graphene (c.a. −4.42 eV) [80], the direct transfer of photogenerated electrons between ZnO and GO could be favorable. This electron transfer might hinder the electron-hole recombination, enhancing the photocatalytic performance under solar irradiation and visible light as previously observed [78,81].

It is noteworthy that amongst all the prepared materials, the composite containing 5.5 wt.% of GO, i.e., ZnO-GO5.5 exhibits not only the best catalytic activity but also the highest TOC removal in comparison with all other composites under solar irradiation and visible-LED (i.e., 57.5 and 20.1%, respectively, Table 2). These results point out the relevance to select the adequate carbon content in the carbon-semiconductor composites in order to obtain the optimal catalytic performance, as previously reported for other graphene-metal oxide composites [58,60]. However, the lower photocatalytic activity observed for the composite containing a GO loading higher than the optimum value (i.e., ZnO-GO6.5 vs. ZnO-GO5.5) could be associated with the scattering effect of GO [77], or the formation of larger aggregates, as shown by SEM (Figure 4), that could hinder the interactions with the irradiation.

In order to evaluate the mineralization under both solar light and visible-LED, the ratio of TOC removal (***X_TOC_***) and VA degradation (***X_VA_***) was introduced in Table 2. In spite of the lower activity obtained under visible-LED compared to solar light (due to the lower extent of the emission spectra), the results show that the experiments under LED illumination were more effective for the mineralization of VA as the ratio (***X_TOC_/X_VA_***) was higher under visible-LED than that obtained under simulated solar light (Table 2). It is also noteworthy that this parameter varies according to the tendency previously observed for the photocatalytic activity, i.e., progressively increases with the increases in GO loading but decreases for GO contents higher than the optimum value (i.e., 6.5 wt.%).

### 3.3. Influence of Hole, Radical, and Non-Radical Scavengers and Study of the Stability

**Figure 9:**
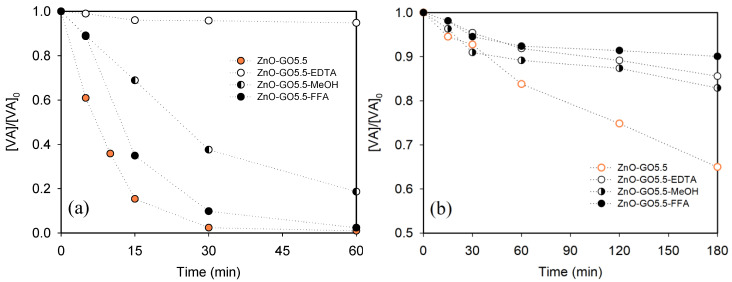
Effect of hole/radical/non-radical scavengers (EDTA/MeOH/FFA) on the photocatalytic degradation of VA using ZnO-GO5.5 under (**a**) simulated solar light and (**b**) visible-LED.

The ZnO-GO5.5 composite was selected for a deep study using hole, radical, and non-radical scavengers, in particular ethylenediaminetetraacetic acid (EDTA), methanol (MeOH), and furfuryl alcohol (FFA), respectively, in order to elucidate the possible reactive oxygen species (ROS) involved in the photodegradation of VA under simulated solar light and visible-LED (Figure 9a,b, respectively).

Figure 9a shows the degradation of VA in presence of the selected scavengers under simulated solar irradiation. The results show that all scavengers used decrease the VA degradation. In particular, this effect was more pronounced in the presence of EDTA and to a lesser extent by MEOH and FFA, obtaining a VA degradation of around 5, 81, and 97%, respectively, compared to 99% with no scavenger. These results indicate that, although reactive radicals (formed from photoexcited electrons) and non-radical species (such as singlet oxygen, ^1^O_2_) are involved in the reaction, the photogenerated holes seem to be the main responsible species in the degradation of VA (by direct oxidation or by formation of radicals such as HO^•^) under simulated solar light.

In the case of visible-LED (Figure 9b), the obtained results also suggest that hole, radical, and non-radical species participate in the reaction mechanism, however, the participation of non-radicals (singlet oxygen, ^1^O_2_ ) in the photocatalytic mechanism seems to play a major representative role in the photocatalytic mechanism under visible-LED, as the degradation observed in presence of FFA was lower in comparison with that obtained for EDTA and MeOH (i.e. 10, 14, and 17%, respectively). The formation of species such as hydroxyl radicals (HO^●^) and hydroperoxyl radicals (HOO^●^) under UV irradiation, as well as the formation of other species such as singlet oxygen (^1^O_2_) and superoxide anion (O_2_^●−^) under visible light, have been reported in the literature as active species during the degradation of contaminants in the aqueous phase using ZnO and other semiconductors [82,83,84,85,86]. These results show the different photocatalytic mechanisms involved in the degradation of water pollutants under simulated solar irradiation and visible illumination [87].

**Figure 10:**
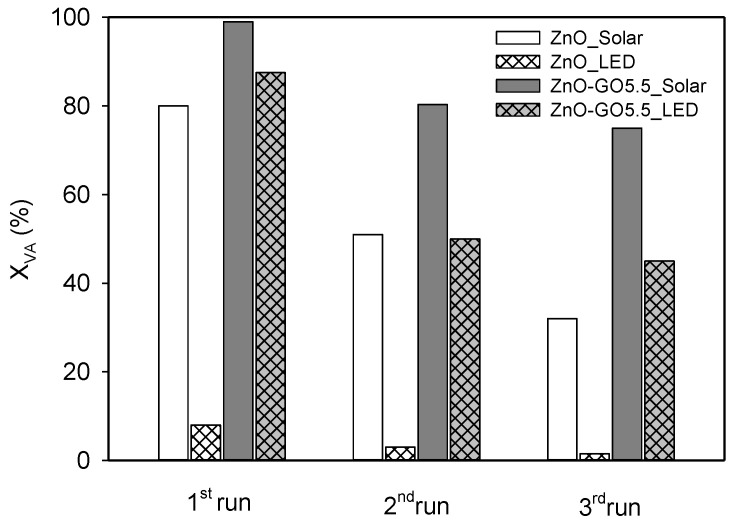
Photocatalytic performance of three consecutive cycles degradation for pristine ZnO and ZnO-GO5.5 under simulated solar light and visible-LED.

The photo-stability of photocatalysts is an important factor for industrial applications. Thus, one of the major drawbacks of ZnO is their severe photo-corrosion under UV/Vis irradiation, which can result in a significant decrease in the photocatalytic activity in reused processes [88,89]. Pristine ZnO and ZnO-GO5.5 were selected in order to evaluate its photocatalytic stability in several recycle runs under simulated solar light (i.e., UV-Vis) and visible-LED for 60 min and 180 min, respectively (Figure 10). The experimental procedure was similar to that described in Section 2.3, but in this case after each reaction, the catalyst was rinsed with water and dried in an oven at 80 °C for 5 h before reuse in two additional consecutive photocatalytic experiments. The photocatalytic activity of pristine ZnO decreased during the three consecutive cycles under solar and LED light. However, stability increased in the case of the composite, the conversion values decreasing slightly after the first cycle under both radiations (Figure 10). In fact, the VA conversion (*X_VA_,* %) was lower in the second run in comparison with the first under both simulated solar light (i.e., from 99 to 80%) and visible-LED (i.e., from 35 to 20%), whereas the photocatalytic activity remained practically constant in the third run (75 and 18% for solar light and visible-LED, respectively) for the composite. These results show that the ZnO-GO composite was stable under solar irradiation and visible-LED, indicating that the photo-corrosion of ZnO could be reduced by the presence of GO. This fact highlights the stability and viability of the prepared ZnO-GO composites to work under continuous mode in future studies.

Table 3 comprises studies regarding different photocatalysts that have been recently published towards phenolic compounds degradation under UV/Vis light irradiation. It can be concluded that the obtained photocatalyst in this work showed a good photocatalytic performance and photostability compared to that of other materials reported in the literature.

## 4. Conclusions

Photocatalysts based on ZnO and GO were prepared with different GO contents. The results indicated a high photocatalytic efficiency for the degradation and mineralization of vanillic acid (VA) under simulated solar light and visible-LED (λ > 430 nm). SEM images showed that pristine ZnO consisted of rod and spherical-like particles. For the composites, more aggregate structures (with the formation of flat assemblies) were observed. The presence of GO in the composites led to superior photocatalytic performance when compared to that obtained from pristine ZnO under solar light and visible-LED. Nevertheless, the highest catalytic performance was obtained with the composite comprising a GO content of 5.5 wt.%, achieving a VA degradation of 99.0% under simulated solar light. Higher *X_TOC_/X_VA_* ratios were obtained under LED light, indicating a more efficient mineralization of contaminant in comparison to that obtained under simulated solar light.

The obtained results from the study of hole, radical, and non-radical scavengers suggested that the photogenerated holes seem to be the main responsible species in the degradation of VA under solar light. The participation of singlet oxygen (^1^O_2_) played a major representative role in the photocatalytic mechanism under visible-LED.

The most effective material, i.e., ZnO-GO5.5, showed better photo-stability in three consecutive runs compared to pristine ZnO, and only a slight decrease of VA degradation was observed under both solar irradiation and visible-LED.

## Figures and Tables

**Table 1:** BET surface area (S_BET_), mesopore volume (V_meso_), total pore volume (V_pore_), pH at the point of zero charge (pH_PZC_), band–gap energy (E_g_) of materials, and crystallite size of synthetized materials.

Samples	*S*_BET_ (m^2^ g^−1^)	*V*_meso_ (cm^3^ g^−1^)	*V*_total_ (cm^3^ g^−1^)	pH_PZC_	E_g_ (eV)	Crystallite Size (nm)
GO	21	n.d.	n.d.	2.8	-	n.d.
ZnO	12	0.07	0.07	7.6	3.12	41
ZnO-GO1.8	19	0.09	0.10	7.4	3.05	36
ZnO-GO2.8	18	0.08	0.09	7.4	3.05	34
ZnO-GO5.5	18	0.13	0.15	7.3	2.98	35
ZnO-GO6.5	10	0.23	0.29	7.3	2.95	35

n.d. = not determined.

**Table 2:** VA conversion (X_VA_), TOC removal (%), pseudo–first order kinetic rate constant (k_ap_), and regression coefficient (r^2^) under simulated solar light and visible-LED.

	Simulated Solar Light (60 Min)					Visible-LED (180 Min)		
Sample	*X_VA_* (%)	*k_ap_* (10^−3^ min^−1^)	r^2^	*X_TOC_* (%)	*X_TOC_/X_VA_*	*X_VA_* (%)	*X_TOC_* (%)	*X_TOC_/X_VA_*
Photolysis	2.8	-	-	-	-	-	-	-
ZnO	85.9	44.7 ± 2	0.992	25.9	0.302	8.1	3.2	0.395
ZnO-GO1.8	90.5	50.2 ± 2	0.993	30.5	0.337	9.9	5.5	0.555
ZnO-GO2.8	93.9	59.4 ± 4	0.988	38.9	0.414	14.4	8.1	0.563
ZnO-GO5.5	99.0	100.8 ± 5	0.995	57.5	0.581	35.0	20.1	0.574
ZnO-GO6.5	93.1	54.6 ± 3	0.991	40.2	0.432	20.7	10.7	0.517

**Table 3:** Compilation of recently published works regarding different photocatalysts for phenolic compounds degradation under UV/Vis light irradiation.

Photocatalyt	Contaminant	Light Source	Catalyst Loading (g L^−^^1^)	Degradation (%)	Ref.
Pd-based photocatalyst	Phenol (20 mg L^−^^1^)	UV-Vis	0.7	93.75	[90]
TiO_2_	Phenolic compounds (0.53 mM)	UV	1	>90	[91]
Au-ZnO nanomaterials	Phenol, catechol and hydroquinone (25 mg L^−^^1^)	UV-Vis	1	>85	[92]
Nano-TiO_2_	Phenolic compounds (100 mg L^−^^1^)	UV	1	97	[93]
rGO-TiO_2_	Phenol, p-chlorophenol and p nitrophenol (20 mg L^−^^1^)	UV and Xenon	0.1	~60	[94]
immobilized nano-ZnO	Phenol (2 mg L^−^^1^)	UV_A_	10–25 m^2^ g^−^^1^	20	[95]
ZnO/TiO_2_-rGO	Phenol (60 mg L^−^^1^)	3 visible Cd lamps	0.6	100	[96]
ZnO-Graphene	Phenol (40 mg L^−^^1^)	Solar radiation	1	>90	[97]
ZnO-GO	Vanillic acid (20 mg L^−^^1^)	Xenon Lamp	1	100	This work
ZnO-GO	Vanillic acid (20 mg L^−^^1^)	LED visible	1	35	This work
